# *BMC Emergency Medicine* reviewer acknowledgement 2014

**DOI:** 10.1186/s12873-015-0029-2

**Published:** 2015-04-08

**Authors:** Giulia Mangiameli

**Affiliations:** BioMed Central, Floor 6, 236 Gray’s Inn Road, London, , WC1X 8HB, , UK

## Abstract

The editors of BMC Emergency Medicine would like to thank all our reviewers who have contributed to the journal in Volume 14 (2014).

**Peter Aitken**

Australia

**Fouad Amraoui**

Netherlands

**Glenn Arendts**

Australia

**Mike Bambach**

Australia

**Caroline Bastiaenen**

Netherlands

**Marian Betz**

USA

**Ulf Björnstig**

Sweden

**Adrian Boyle**

UK

**Georges Brousse**

France

**Enrique Casalino**

France

**Maaret Castren**

Sweden

**Razvan Chereches**

Romania

**Corinne Chmiel**

Switzerland

**Tae Nyoung**

Chung South Korea

**Federico Coccolini**

Italy

**Rafael Consunji**

Qatar

**Sharmila Dissanaike**

USA

**Jhodie Duncan**

Australia

**Marina Economou**

Greece

**Russell Griffin**

USA

**Joerg Haasenritter**

Germany

**Roman Haberl**

Germany

**Jin Han**

USA

**Alys Havard**

Australia

**Brian Hiestand**

USA

**Matthew Hopcraft**

Australia

**Jan Jensen**

Canada

**Iolanda Jordan**

Spain

**Narasimhan Kannan**

Singapore

**Anita Kar**

India

**Carol Kotliar**

Argentina

**Erik Kulstad**

USA

**Emmanuel Lagarde**

France

**Timo Lajunen**

Norway

**Eddy Lang**

Canada

**Rifat Latifi**

USA

**Carl Leier**

USA

**Resa Lewiss**

USA

**Maxime Maignan**

France

**Marek Majdan**

Slovakia

**Sheryl Martin**-**Schild**

USA

**Robert Maunder**

Canada

**Walter Mauritz**

Austria

**Russell Mcculloh**

USA

**Amee Morgans**

Australia

**Dan Mullany**

Australia

**Romesh Nalliah**

USA

**Theresa Mariero Olasveengen**

Norway

**Jake Olivier**

Australia

**Sukhmeet Panesar**

UK

**Asad Patanwala**

USA

**Carmen Andrea Pfortmueller**

Switzerland

**Timothy Platts**-**Mills**

USA

**Douwe Postmus**

Netherlands

**Colin Powell**

UK

**Michael Puskarich**

USA

**Nithima Ratanasit**

Thailand

**Marius Rehn**

Norway

**Joshua Reynolds**

USA

**Schalk Richard**

Germany

**Antoine Roch**

France

**Sten Rubertsson**

Sweden

**Jan Schieveld**

Netherlands

**Farshad Shirazi**

Algeria

**Dimitrios Siassakos**

UK

**Lorraine Smith**

Australia

**Eldar Soreide**

Norway

**Leandro Souza**

Brazil

**Tracy Stecker**

USA

**Christopher Stevenson**

Australia

**Melanie Taylor**

Australia

**Marc Tennant**

Australia

**Carol B Thompson**

USA

**Ebbe Billmann Thorgersen**

Norway

**Hans Thulesius**

Sweden

**James Tsung**

USA

**Cynthia Van Der Starre**

Netherlands

**Carmen Vleggeert**-**Lankamp**

Netherlands

**Johan Von Schreeb**

Sweden

**Marko Vujicic**

USA

**Scott Weinstein**

Australia

**Leana Wen**

USA

**Derek Wheeler**

USA

**Marcel Wolbers**

Vietnam

**Nan**-**Ping Yang**

Taiwan

**Sa**‘**Ed Zyoud**

Palestinian Territory

